# Anti-inflammatory drug-eluting implant model system to prevent wear particle-induced periprosthetic osteolysis

**DOI:** 10.2147/IJN.S188193

**Published:** 2019-02-08

**Authors:** Melissa C Rivera, Stefano Perni, Alastair Sloan, Polina Prokopovich

**Affiliations:** 1 School of Pharmacy and Pharmaceutical Sciences, Cardiff University, Cardiff, UK, prokopovichp@cf.ac.uk; 2 School of Dentistry, Cardiff University, Heath Park, Cardiff, Wales, UK

**Keywords:** titanium, dexamethasone, macrophages, aseptic loosening, TJR, implant

## Abstract

**Background:**

Aseptic loosening, as a consequence of an extended inflammatory reaction induced by wear particles, has been classified as one of the most common complications of total joint replacement (TJR). Despite its high incidence, no therapeutical approach has yet been found to prevent aseptic loosening, leaving revision as only effective treatment. The local delivery of anti-inflammatory drugs to modulate wear-induced inflammation has been regarded as a potential therapeutical approach to prevent aseptic-loosening.

**Methods:**

In this context, we developed and characterized anti-inflammatory drug-eluting TiO_2_ surfaces, using nanoparticles as a model for larger surfaces. The eluting surfaces were obtained by conjugating dexamethasone to carboxyl-functionalized TiO_2_ particles, obtained by using either silane agents with amino or mercapto moieties.

**Results:**

Zeta potential measurements, thermogravimetric analysis (TGA) and drug release results suggest that dexamethasone was successfully loaded onto the TiO_2_ particles. Release was pH dependent and greater amounts of drug were observed from amino route functionalized surfaces. The model-system was then tested for its cytotoxic and anti-inflammatory properties in LPS-stimulated macrophages. Dexamethasone released from amino route functionalized surfaces TiO_2_ particles was able to decrease LPS-induced nitric oxide (NO) and TNF-a production similarly to pure DEX at the same concentration; DEX released from mercapto route functionalized surfaces was at a too low concentration to be effective.

**Conclusion:**

Dexamethasone released from amino functionalized titanium can offer the possibility of preventing asepting loosening of joint replacement devices.

## Introduction

Joint degenerative and inflammatory diseases, eg, arthritis, affect the quality of life of millions of people worldwide.^1^ One of the ways to help people affected by such disease is to have artificial implants such as total joint replacement (TJR).^2^–^4^ Even though the success of these procedures has been widely recognized, so far it is not yet possible to overcome wear debris-induced osteolysis, a process that leads to aseptic loosening. TJR-associated osteolysis is difficult to treat and, in most of cases, requires complete removal of the implant. This procedure is costly and time-consuming for both patients and health services due to its complexity, high risk, and often suboptimal surgical outcome.^5^ According to National Joint Registry for UK, 9% of hip and 6% of knee primary procedures undertaken in UK during 2015 resulted in revision surgery. Of those recorded, aseptic loosening was the cause for total hip and knee revision procedures in 42% and 41% of cases, respectively.^6^

Although different theories have been proposed to explain TJR aseptic loosening,^7^ it is generally accepted that the factors leading to osteolysis involve the release of wear particles originated during the articulation of the joint device.^8^,^9^ Wear debris triggers an inflammatory reaction, which leads to osteoclast differentiation and osteoblast downregulation, thus ultimately resulting in local bone (osteolysis) loss around the implant.

In order to overcome such adverse events, research has concentrated on methods to enhance osseointegration and diminish the release of wear particles in orthopedic implants.^2^,^9^,^10^ For this purpose, alternative bearing materials, such as cross-linked polyethylene, zirconium/zirconia combination, titanium- and cobalt-based alloys, and polycarbonate-urethane, have been suggested.^11^,^12^ Also the application of surface treatments such as plasma spraying, grit blasting, acid etching, and electrochemical anodization has been used to mitigate the risk of osteolytic reaction.^2^,^13^,^14^ However, some of these methods have been reported to affect the physical integrity of the implant, hence causing adverse effects on surrounding cells and tissues.^15^,^16^

A more recent approach to address wear debris-induced aseptic loosening is the direct modulation of the inflammatory response by using implant surfaces eluting anti-inflammatory drugs.^17^,^18^ The main advantage of these systems is the local delivery of anti-inflammatory molecules, which simultaneously modulate inflammation and avoid possible side effects caused by a long-term and high concentration systemic drug administration, most notably the gastrointestinal and cardiovascular adverse effects.^19^ Implantable biomaterials and devices with different anti-inflammatory delivery systems have been reported in literature, such as bone cements,^20^ nanotubes,^21^,^22^ dendrimer conjugates,^23^,^24^ *N*-(2-hydroxypropyl) methacrylamide copolymer,^17^,^25^ and poly(lactic-co-glycolic acid) microspheres.^26^ Besides anti-inflammatory activity,^27^ these systems have also shown several advantages in orthopedic applications such as improved biological activity in terms of osteoblasts attachment, proliferation, and differentiation.^28^,^29^ Although the utilization of anti-inflammatory drug-eluting implants has shown some significant improvements, most of them are still in early research stage.

Particularly, in most of the drug delivery systems described in literature, the anti-inflammatory drug is released in uncontrolled manner, ie, burst release and within the first week after implantation. This short-timed and uncontrolled release is not long enough to modulate the acute phase of inflammation, which can last for >1 week. Long acute inflammation can possibly result in chronic inflammation, which is characterized by poor tissue healing that creates mechanical instability around the implant. This leads to the conclusion that no effective solutions to avoid or treat aseptic loosening have been found yet. Therefore, it is evident that the development of novel and better approaches to modulate wear debris-induced inflammatory response is needed.

This study aims to develop an anti-inflammatory drug-eluting model system of titanium surface to modulate the wear debris-induced inflammation, which has been elucidated as the central mechanism for aseptic loosening. Due to its cost-effective characteristics, high surface area, and easy scale-up,^30^,^31^ TiO_2_ particles have been utilized as a drug delivery model system. It is also worth noting that the surface modifications performed on TiO_2_ particles could be conducted on the surface of clinically used implants made of titanium and titanium alloys, which are regarded as one of the most widely used biomaterials in joint replacements.^32^ Therefore, the model system may serve as proof of concept to incorporate anti-inflammatory drugs in implant materials for TJRs made of titanium and titanium alloys. In addition, dexamethasone (DEX), a synthetic glucocorticoid,^33^ with its high potency and effectiveness against inflammation,^34^ has been chosen as anti-inflammatory drug model. We hypothesize that DEX release at bone–implant interface will attenuate the inflammatory response induced by wear debris without causing the adverse side effects reported for oral administration, eg, gastrointestinal bleeding and perforation.^35^ We further hypothesize that, by preparing TiO_2_ particles loaded with DEX through different synthetic routes, different level of drug loading, different release kinetics, and subsequently different anti-inflammatory efficiency will be obtained. Considering these hypothesis, TiO_2_ particles were prepared by modifying the surface of TiO_2_ through amino and mercapto synthetic routes for subsequent attachment of DEX.

One of the hallmarks of inflammation is the recruitment and activation of macrophages^36^,^37^ that produce a variety of inflammatory mediators, eg, ILs, prostaglandins, and reactive species such as highly reactive free radicals and nitric oxide (NO).^38^,^39^ To assess the anti-inflammatory properties of the model system developed, we evaluated the effect of DEX released from TiO_2_ particles on cytotoxicity and lipopolysaccharide (LPS)-induced NO and tumor necrosis factor-a (TNF-a) production in a macrophage-like cell line (RAW 264.7) and compared its performance with dexamethasone phosphate (DEX-P) that is currently used in clinical practice.

## Experimental

### Chemicals

Titanium (IV) oxide (anatase, <25 nm), (3-mercaptopropyl)trimethoxysilane (MPTMS, 95%), (3-aminopropyl) triethoxysilane (APTES, 99%), succinic anhydride (≥99%), DEX (>97%), 4-pentenoic acid (≥98%, FG), 1-(3-dimethylaminopropyl)-3-ethylcarbodiimide hydrochloride (EDC, +98%), N-hydroxysulfosuccinimide (Sulfo-NHS, 98%), N-morpholino ethanesulfonic acid (MES) hydrate (>99.5%), PBS tablets, sodium acetate trihydrate (≥99%), sodium chloride (ACS reagent, ≥99%), citric acid monohydrate (ACS reagent, ≥99%), and disodium phosphate (ACS reagent, ≥99%) were purchased from Sigma-Aldrich, Haverhill, UK. HPLC grade acetonitrile, glacial acetic acid, methanol, dichloromethane (DCM), and toluene were purchased from Fisher, Loughborough, UK. All other chemicals were analytical reagent grade stored according to manufacturer’s guidelines and used as received.

### DEX-functionalized synthesis of TiO_2_ particles

**Figure 1:**
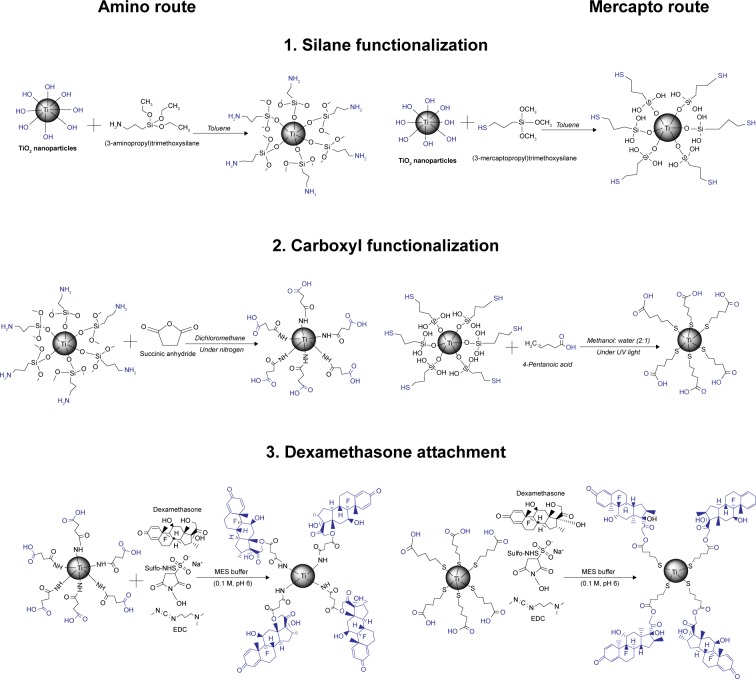
Detailed schemes of chemical reactions for dexamethasone-TiO_2_ particles: 1) silane functionalization, followed by 2) carboxyl functionalization, and then 3) dexamethasone attachment, for amino and mercapto routes. Functional groups on TiO_2_ particle surface are highlighted with blue color.

The synthesis of DEX-TiO_2_ particles was achieved using a two-step surface functionalization process; first, the TiO_2_ particles were surface modified using silane agents, followed by carboxyl functionalization (Figure 1). The second step was the DEX attachment based on the covalent bond formation between the carboxylic acid groups on functionalized TiO_2_ particles (TiO_2_–COOH) and the DEX-hydroxyl groups. Surface modification with silane agents and carboxyl functionalization steps are different for each route, whereas the step of DEX conjugation was the same for amino- and mercapto-functionalized TiO_2_ particles.

#### Surface functionalization

In a typical synthesis, 1 g of TiO_2_ particles was dispersed in 15 mL of anhydrous toluene. The TiO_2_ particle surface was functionalized with amine or mercapto groups by adding 100 µL of either APTES (amino route) or MPTMS (mercapto route), respectively, under stirring and incubating for 24 hours. Subsequently, the functionalized particles (TiO_2_–NH_2_ or Ti–SH) were isolated by centrifugation at 14,000 rpm for 5 minutes (LE-80K Ultracentrifuge, Beckman Coulter, Royston, UK) at 20°C followed by washing for at least three cycles. The resulting particles were dried under the fume hood for 24 hours.

Amine-functionalized particles (TiO_2_–NH_2_) (250 mg) were dispersed in 100 mL of DCM and purged with nitrogen. Succinic anhydride (25 mg) was dissolved in 2.5 mL of DCM and added to the reaction mixture to obtain carboxylic acid-functionalized surface. This suspension was kept under nitrogen for 18 hours under constant stirring. Subsequently, the succinylated particle suspension was separated by centrifugation at 14,000 rpm for 5 minutes at 20°C (LE-80K Ultracentrifuge, Beckman Coulter) followed by washing for at least three cycles with methanol to remove unreacted materials. The resulting particles were dried under the fume hood for 24 hours.

Thiol-functionalized (Ti–SH) particles (250 mg) were dispersed in 100 mL of methanol:water (2:1) solution and 4-pentenoic acid (25 mg) was added. This suspension was kept under ultraviolet using a UV lamp (UVGL-58, Cambridge, UK), λ=254 nm for ~3 hours. The particles were separated by centrifugation at 14,000 rpm for 5 minutes at 20°C (LE-80K Ultracentrifuge, Beckman Coulter) followed by washing for at least three cycles with methanol to remove unreacted materials. The resulting particles were dried under the fume hood for 24 hours.

#### DEX loading

Fifty milligrams of DEX were dissolved in 100 mL of MES buffer (0.1 M, pH 6.0). This solution was used to disperse 250 mg of carboxyl-functionalized particles (amino or mercapto route), 10 mg EDC, and 10 mg Sulfo-NHS. The suspension was kept under vigorous mixing for 24 hours at room temperature. DEX-loaded particles were recovered by centrifugation at 14,000 rpm for 5 minutes at 20°C by washing for at least three cycles. Then, DEX-loaded particles were left to dry under the fume hood for 24 hours.

### Particle characterization

#### Transmission electron microscopy (TEM)

Images of particles were obtained using a Zeiss 902 TEM operating at a voltage of 80 kV. The aqueous dispersion of the particles was dropcast onto carbon-coated copper grids, and grid was dried at room temperature (20°C) before loading into the microscope (direct deposition). The average particle size, size distribution, and morphology analysis of the samples were carried out from transmission electron micrographs using ImageJ for Windows (version 1.50i).

#### Zeta potential measurements

The electrophoretic mobility was determined by dynamic light scattering with a Malvern Zetasizer, Nano ZS particle characterization system (Malvern Instruments Limited, Royston, UK). Zeta potential values were calculated by Smoluchowski’s relation/approximation, converting electrophoretic mobility to zeta potential values. The measurements were performed in a folded capillary cell. For measuring zeta potential, 1 mg of particles was dispersed in 1 mL of buffer solution using the vortex and the ultrasonic bath (~30 minutes), and then the suspension was immediately transferred into the capillary cell. For pH 4, 5, and 6, an acetic acid/sodium acetate buffer (0.01 M) was used. As for pH 7, sodium chloride (NaCl) buffer (0.01 M) was utilized. Results are given as the average ± SD of three measurements on three independent replicates.

#### Thermogravimetric analysis (TGA)

TGA was performed using a Perkin-Elmer TGA 4000 instrument. The samples were heated from 50°C to 800°C with a constant heating rate of 10°C/min. Weight loss percentage of each sample was calculated relative to the initial sample weight prior to heating. As for organic material content was calculated as weight loss (%) when the line plateaus (approximately around 800°C), while inorganic matter was assumed to be the remaining particle component.

#### Fourier-transformed infrared (FTIR)

FTIR spectra were recorded on a Shimadzu IRAffinity-1S apparatus equipped with ATR diamond accessory, at room temperature between 4,000 and 500 cm^−1^ with 4 cm^−1^ resolution running 16 scans.

### DEX release profile

DEX-loaded particles were dispersed (10 mg/mL) in citric acid-disodium phosphate buffer (0.1 M) at pH ranging from 4 to 7. Samples were incubated statically at 37°C, and every 24 hours the particles were separated from the suspension, thus the supernatant was collected to analyze DEX release and redispersed in fresh buffer. The amount of DEX released was quantified using reversed-phase HPLC system with 1,100 series Agilent Technologies HPLC. The mobile phase was a mixture of PBS:acetonitrile:glacial acetic acid 70:26:4 at a fixed flow rate of 1 mL/min. Samples of 10 µL were injected and detected by UV at 244 nm. The analytical column was Water Spherisorb ODS2 column (pore size = 80 Å, 5 µm, and width × length = 4.6 mm × 150 mm). To evaluate the concentration of DEX in the samples collected, a calibration curve of the HPLC detection of DEX was previously obtained. All experiments were performed in triplicate.

### Cell culture and treatments

RAW 264.7 cells were obtained from European Collection of Cell Cultures. Cells were grown in DMEM containing 10% FBS and 100 U/mL penicillin/streptomycin, under 5% CO_2_ at 37°C, in humidified air. Cell numbers and viability were assessed by the trypan blue dye exclusion method. Cells were seeded at a density of 2×10^4^ cells/well in a 96-well plate and permitted to adhere over a 4- to 5-hour period. Cells were then treated with DEX-P (3.9 µg/mL) or elutes/broths of functionalized TiO_2_ particles collected from drug release studies (sterilized through filtering) containing the same amount of DEX. The concentration of DEX-P was determined in preliminary experiments as that capable of reducing inflammation without affecting viability (Supplementary material, S1–S6). DEX-P solutions were prepared at 39 µg/mL and release buffers were diluted to the same concentration; these solutions were then added to the cell medium in a ratio 1:10 to reach the final concentration of 3.9 µg/mL. Because the maximum concentration of DEX achieved from the mercapto route was not enough to allow this dilution and cells did not grow/survive higher concentrations of buffers, these particles were not investigated further.

After incubation for 1 hour, LPS (1 µg/mL) was added to stimulate the macrophages and they were cultured for up to 3 days. Biological responses (cell viability, NO, and TNF-a production) were assessed at different time points (18, 24, 48, and 72 hours). The effect of DEX in the absence of LPS was also assessed to observe whether it induced changes in basal levels of assays performed.

### Biological assays

#### MTT assay

Cellular viability/proliferation of cells after being exposed to DEX was assessed using the mitochondria-dependent reduction of MTT to formazan. After incubation with DEX, the culture medium was removed and 20 µL of the MTT solution (5 mg/mL) was added to each well and incubated at 37°C in a humidified 5% CO_2_ atmosphere for another 4 hours. The supernatant was then carefully discarded, and 100 µL of dimethyl sulfoxide was added to each well to solubilize formazan. The absorbance at 540 nm was measured using ELISA reader. Results were expressed as a percentage of the respective control (with or without LPS).

#### Measurement of NO levels

Hundred microliters of cell culture supernatant was mixed with the same volume of Griess reagent (Thermo Fisher Scientific) for 30 minutes at room temperature. Then, the nitrite produced was determined by measuring optical density of the mixture at 548 nm in a microplate reader. Nitrite was quantified by an external standard, using potassium nitrate. Results are expressed as a percentage of the respective control (with or without LPS).

#### Measurement of TNF-a levels

TNF-a released by macrophage cells were determined using the appropriate ELISA kit (Sigma, Dorset, UK) following the manufacturer’s recommended protocol.

### Statistical analysis

Unless stated otherwise, all the tests described above were performed with at least three independent experiments (each experiment had three replicates) and all data were subject to a one-way ANOVA to assess the statistical significance of results between groups. Experimental results were considered statistically significant at 95% confidence level (*P*<0.05). Tukey’s multiple comparison post hoc test was performed between all groups. All analyses were run using the SPSS software.

## Results and discussion

In this study, a DEX delivery system was developed through the surface functionalization of TiO_2_ particles using different approaches, amino and mercapto. The utilization of different routes was applied to assess if different linkers, ie, functional groups between DEX and TiO_2_ particles, could affect the loading efficiency of DEX, drug release profile, and also cellular behavior, eg, toxicity, bioactivity. Surface functionalization, DEX loading efficiency, zeta potential measurements, and TGA analysis were performed to provide evidence of different organic groups attached, ie, –NH_2_ and –SH on the surface of TiO_2_ particles, including DEX loading.

### Characterization of DEX-loaded TiO_2_ particles

**Figure 2:**
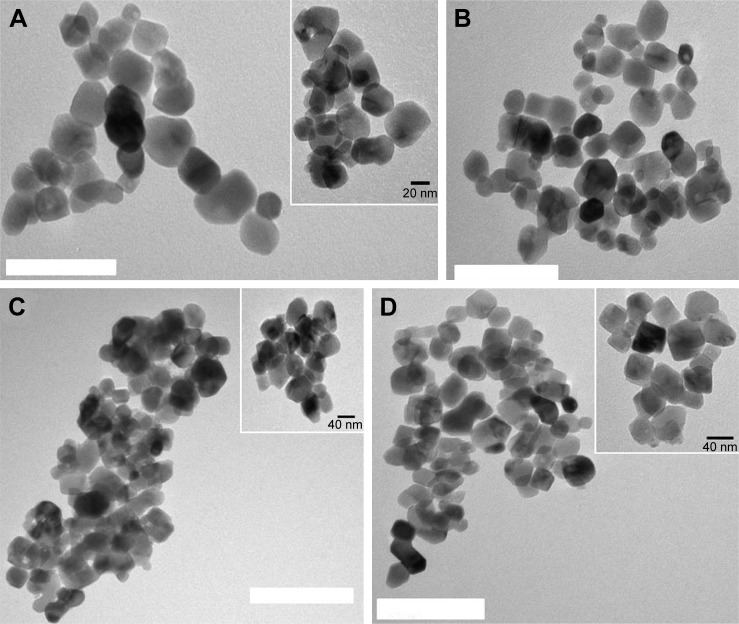
Examples of transmission electron microscopy images of different surface-modified TiO_2_ particles: (**A**) TiO_2_ particles, (**B**) amino-functionalized, (**C**) succinylated amino-functionalized, and (**D**) DEX-loaded TiO_2_ particles. Bar represents 100 nm.

Determination of morphology and average size Examples of TEM images of the uncoated TiO_2_ particles and functionalized ones (Figure 2) were taken to determine the shape/structure and size of the particles after each step of functionalization. The radius of unfunctionalized TiO_2_ particles was around 20–25 nm with a narrow size distribution. Moreover, they showed a cubical and spherical-like shape with dense core, which is characteristic of TiO_2_ nanoparticles. After each step of functionalization (amino, carboxyl, and DEX loading), the size of particle diameter remained around 25–30 nm, without obvious morphological changes. A similar trend was observed for mercapto route functionalization particles (shown in the Supplementary material).

Overall, images obtained via TEM revealed that for all synthetic routes tested the different steps of TiO_2_ particle functionalization did not damage the particle structure. It also revealed that the thickness of the functionalizing layer was negligible.

#### Zeta potential measurements

**Figure 3:**
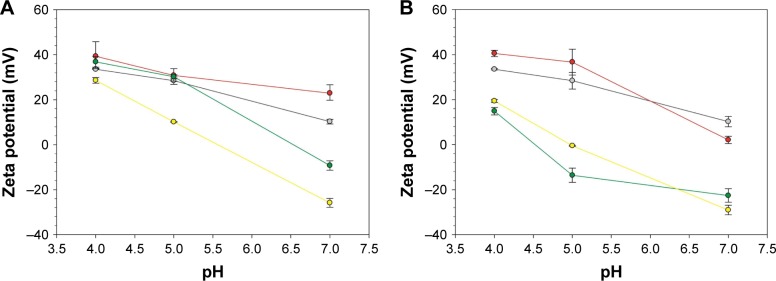
Zeta potentials of different surface-modified TiO_2_ nanoparticle suspensions at different pH values for amino (**A**) route: TiO_2_ bare NP (−); amino-functionalized TiO_2_-NH_2_ (−); succinylated amino-functionalized TiO_2_-COOH (−); DEX conjugated to succinylated amino-functionalized TiO_2_-COOH (−); and for mercapto (**B**) route: TiO_2_ bare NP (−); mercapto-functionalized TiO_2_-SH (−); carboxyl mercapto-functionalized TiO_2_-COOH (−); DEX conjugated to succinylated mercapto-functionalized TiO_2_-COOH (−). **Abbreviations:** DEX, dexamethasone; NP, nanoparticle.

Zeta potential measurements were used to assess the attachment of organic groups, ie, –NH_2_, –SH, and –COOH, after each step of TiO_2_ particle functionalization, including DEX loading (Figure 3). As pH becomes more alkaline, a decrease on surface potential was observed; on the contrary, the highest surface potentials were observed under acidic pH. A more positive surface charge was observed after amino and mercapto functionalization when compared with bare TiO_2_ particles. On the contrary, carboxyl-functionalized and DEX-loaded TiO_2_ particles showed a more negative surface charge than amino- and mercapto-functionalized particles.

These results can be explained based on the ability of protons to neutralize negatively charged groups at low pH values. The positive surface charge observed after amino functionalization of TiO_2_ particles can be related to the amino groups (–NH_2_) ability of accepting protons, becoming positively charged (NH_3_^+^). On the contrary, the decrease of zeta potential under alkaline conditions (pH >6) can be attributed to the deprotonation of the amino group on the surface of TiO_2_ particles. In contrast, lowest surface potentials were observed for succinic anhydride and pentanoic acid TiO_2_ particles, especially for more alkaline pH values. A possible explanation for this character could be attributed to the presence of carboxylic acid (–COOH) groups on surface of the particles, which exhibit a more negative character than NH_2_– and SH–TiO_2_ particles. The same behavior, ie, more negative surface potential, was observed after DEX loading, for both mercapto and amino route. The higher negative zeta potential observed might be attributed to the presence of functional groups (–OH and –C=O) of DEX, which are on the surface of the TiO_2_ particles. In particular, the fluorine group (9-Fluoro) has been described to affect the electron density of its “neighbors”, ie, functional groups.^50^ In this specific case, fluorine seems to be responsible for decreasing the pKa value of hydroxyl moiety (11, 17, 21-trihydroxy) on DEX, thus resulting in a more negative zeta potential after DEX loading. Similar results were described by Fratoddi et al that obtained a negative zeta potential after loading particles with DEX under alkaline conditions, ie, aqueous particle suspension.^51^

#### Thermogravimetric analysis

**Figure 4:**
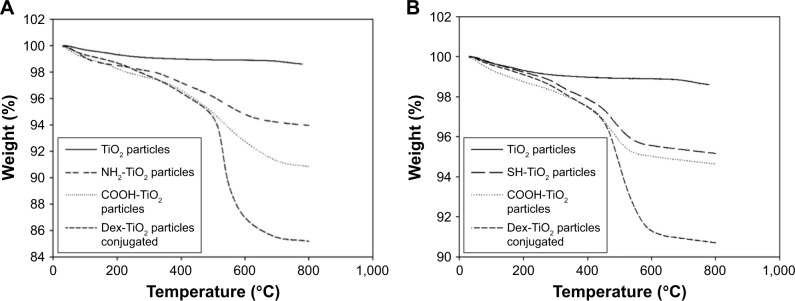
Thermograms of different surface-modified TiO_2_ particles prepared through amino (**A**) and mercapto route (**B**).

The TGA profile of TiO_2_ particles after each functionalization step revealed an initial weight loss (~1%) at about 100°C (Figure 4), which is normally attributed to the evaporation of adsorbed water from the samples. Due to this reason, the organic content of each sample (Table 1) was calculated based on the weight loss beyond 100°C, which truly corresponds to the combustion of organic species. It was also observed that, after each functionalization step, there was an increased amount of organic content on the surface of TiO_2_ particles.

Results from TGA analysis (Table 1 and Figure 4) showed an increase of organic content after amino and carboxyl functionalization when compared with organic content of TiO_2_ particles (1.5%); 6.0% for amino-TiO_2_ particles and 9.2% for carboxyl-functionalized TiO_2_ particles. Moreover, the same behavior was observed for DEX-loaded particles, where an increase of organic content of ~6% was detected when compared with carboxyl-functionalized TiO_2_ particles.

An increase of organic content was observed after mercapto and carboxyl functionalization when compared with organic content of TiO_2_ particles (1.5%), 4.8% for mercapto-TiO_2_ particles and 5.4% for carboxyl-functionalized TiO_2_ particles. Moreover, the same behavior was observed for DEX-loaded particles, where an increase of organic content to ~9.2% was detected when compared with carboxyl-functionalized TiO_2_ particles (5.4% of organic content).

TGA profile obtained was similar and in agreement to the one obtained by Bach et al.^40^ The TGA profile of particles obtained via amino and mercapto route (Figure 4) showed an increase of organic material of ~3.1% after APTES and 2.6% after (3-mercaptopropyl)triethoxysilane (MPTES) functionalization, thus confirming the successful attachment of amino and mercapto functional groups on the surface of TiO_2_ particles.

The lower load achieved through the mercapto route could be attributed to the lower efficiency in the reaction between the SH groups and the pentanoic acid as seen in both the TGA and the FTIR analysis; as this route gave fewer COOH groups on the surface, it can be expected that the conjugation of DEX would result in fewer molecules of the drug attached.

#### FTIR spectral studies

**Figure 5:**
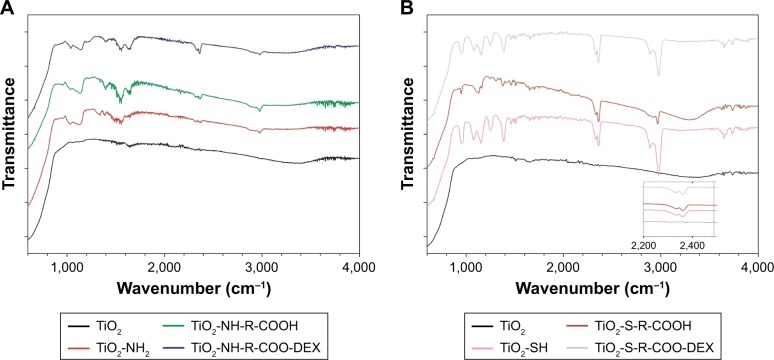
FTIR spectra of TiO_2_ bare NP (−); amino-functionalized TiO_2_-NH_2_ (−); succinylated amino-functionalized TiO_2_-COOH (−); DEX conjugated to succinylated amino-functionalized TiO_2_-COOH (−); mercapto-functionalized TiO_2_-SH (−); carboxyl mercapto-functionalized TiO_2_-COOH (−); DEX conjugated to succinylated mercapto-functionalized TiO_2_-COOH (−). **Abbreviations:** DEX, dexamethasone; FTIR, Fourier-transformed infrared; NP, nanoparticle.

The spectra of functionalized and untreated TiO_2_ particles are shown in Figure 5. FTIR spectrum of untreated TiO_2_ nanoparticles showed a broad peak appearing at 3,100–3,600 cm^−1^ due to vibrations of hydroxyl groups.^40^ The absorption peak around 1,620 cm^−1^ was caused by bending vibration of coordinated H_2_O as well as from the hydroxyl groups on TiO_2_ surface.^41^ Moreover, the intense broadband in the vicinity of 800 cm^−1^ was attributed to the stretching vibration of Ti–O–Ti in anatase morphology.^42^ Compared with the spectra for untreated TiO_2_ particles, a new peak located at around 1,360 cm^−1^ in the spectra of NH_2_–TiO_2_ particles appeared, which can be assigned to carbon–nitrogen bonds. The presence of the previous described bands and peaks on the FTIR spectrum of amino-functionalized and untreated TiO_2_ particles are in reasonable agreement with those reported in the literature.^43^ The COOH–TiO_2_ particle spectra showed a new peak around 1,690 cm^−1^, indicating the presence of –C=O stretching vibration band.^44^ These findings suggested that carboxyl groups were present onto the surface of the particles after successful succinylation.

When comparing bare TiO_2_ particles with those functionalized with mercapto-silane (MPTES), the band corresponding to thiol (SH) groups could be clearly observed at around 2,400 cm^−1^ (inset Figure 5B). Moreover, the bands at ~3,000 and 2,800 cm^−1^ attributed to the C–H stretch of methylenes of the alkyl chain were obvious, suggesting that the new functional groups were successfully attached to the surface of TiO_2_ particles.^45^

The other visible peaks between 900 and 1,100 cm^−1^ are due to Si–O–Si and Si–O stretching vibrations; additionally, the O–H broad peak (3,100–3,600 cm^−1^) that appeared in the spectrum of bare TiO_2_ particles was less visible, which coincides with a decrease in the hydroxyl groups due to the attachment of the MPTES.^46^

As for carboxyl and DEX functionalization, the FTIR equipment was not sensitive enough to detect the characteristic peaks/bands. Although different FTIR spectra were observed, particularly a broad peak between 3,100 and 3,600 cm^−1^ was visible, which coincides with the increased presence of the hydroxyl groups (O–H) due to the attachment of COOH functional and OH groups on surface of functionalized TiO_2_ particles.

### DEX release profile

**Figure 6:**
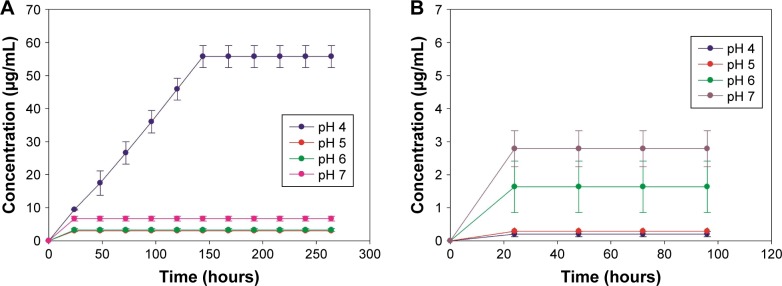
Cumulative release of dexamethasone from TiO_2_ particles obtained via amino route (**A**) and mercapto route (**B**) under different pH conditions.

In vitro release studies were performed under different joint conditions, healthy joints (pH =~7),^47^ and inflamed joints, which are associated with local acidosis.^48^ For most of the conditions tested (pH =5, 6, and 7), DEX was released within the first 24 hours (Figure 6). Although, at pH =4, DEX release from TiO_2_ particles obtained via amino route was prolonged, where the drug was constantly released for 6 days. Moreover, for both amino and mercapto particles, the drug release was pH-dependent; for particles obtained via amino route, concentration of DEX increases with decreasing pH. Oppositely for particles obtained via mercapto route, the highest release of DEX was obtained under physiological conditions (pH =7).

Because the maximum concentration of DEX achieved from the mercapto route was not sufficient, these particles were not investigated further (for the biological assays).

DEX release profile was expected to mainly depend on the structure and hydrolysis rate of the linker between DEX and TiO_2_ particles,^52^ both of which are strongly affected by the type of synthetic route, ie, amino and mercapto, utilized in this study.

Results show that for particles obtained via amino route (Figure 6A), DEX release kinetics were faster under the most acidic conditions (pH =4); moreover, in this condition, release continued over a period of 6 days, a behavior which was only observed for this pH value. DEX was conjugated to the TiO_2_ particles through ester bonds; hence, its release was due to hydrolysis of this bond. The rate of this reaction increases further from neutrality, thus leading to the gradually greater release of DEX from pH =7 to pH =4. A similar drug release profile was observed by Nuttelman et al,^52^ where DEX was covalently attached to polyethylene glycol-based hydrogel scaffold and for gentamicin when attached to gold^53^ and silica.^49^

As for mercapto route (Figure 6B), results showed that DEX presented a less efficient, and with lower drug concentrations, release when compared with amino route. Furthermore, when comparing the overall drug released from amino and mercapto particles, it can be easily seen that a higher amount of DEX was released when amino route was used. This drug release profile is in agreement with TGA analysis, which reveals that a higher percentage of organic content was observed after DEX attachment to amino-TiO_2_ particles when compared with the ones obtained via mercapto route (Table 1). However, despite DEX loading achieved through the mercapto route that was only marginally lower than the amino route, the release was considerably lower (Figure 6). This is likely due to the more difficult hydroxylation of the ester bond between DEX and functionalized surface when the mercapto route was used. The ester bond is the same in both amino and mercapto routes, although the number of carbon differs: four for amino route and three for mercapto route (Figure 1). This variation in the number of carbon groups could also explain the more efficient release of DEX when amino route was applied, where the longer the linker the easier the hydrolysis of the ester bond as more assess required by nucleophile compounds to initiate hydrolysis is less sterically hindered.

The time frame aspects of anti-inflammatory drug release that will modulate the foreign body response to implant are still not well understood. Although it is known that to obtain an efficient modulation of inflammatory response, the release of drug should at least be continuous.^55^ Taking into account that the inflammatory tissue response starts after the first second after device implantation,^54^ the immediate DEX release, ie, within first 24 hours observed from both mercapto and amino TiO_2_ particles, could be highly favorable to control initial acute inflammation (1–3 days) at the site of implantable devices.^55^

### Cellular viability studies

**Figure 7:**
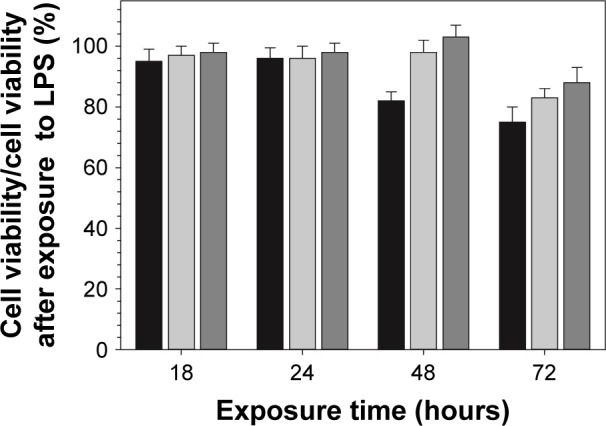
Effect of DEX on cell viability of LPS-activated RAW 264.7. **Notes:** Cells were exposed to DEX (3.9 µg/mL) either from filtered broth collected after the first 24 hours of release (black columns) or DEX-P for 18, 24, 48, and 72 hours (light gray columns). Cellular viability was assessed by MTT assay, and 10% PBS buffer was used as positive control (dark gray columns). **Abbreviations:** DEX-P, dexamethasone phosphate; LPS, lipopolysaccharide.

Results obtained from MTT assay showed that the viability of cells exposed to DEX released from functionalized particles obtained through amino route for different periods of time was very similar (>90% cellular viability) to the one observed for the same concentration of pure DEX administered as DEX-P (Figure 7).

### Anti-inflammatory activity of DEX-loaded TiO_2_ particles

The anti-inflammatory activity of DEX released from the model system (DEX-loaded functionalized TiO_2_ particles) obtained by amino synthetic route was compared with the anti-inflammatory activity of same concentration of pure DEX administered as DEX-P.

**Figure 8:**
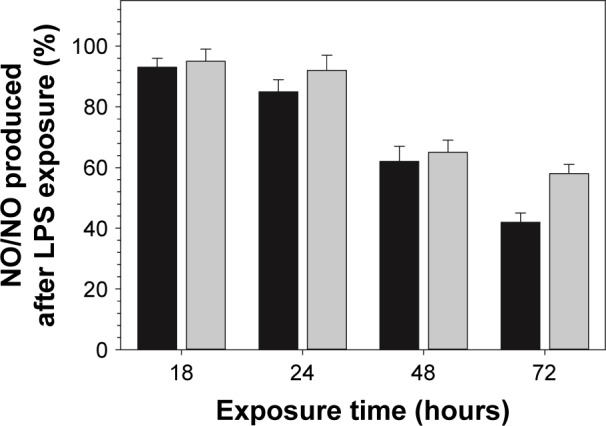
Effect of DEX on NO production (percentage to NO in LPS-only control) of LPS-activated RAW 264.7. **Notes:** Cells were exposed to DEX (3.9 µg/mL) from filtered broth collected after the first 24 hours of release (black columns) or DEX-P (DEX equivalent 3.9 µg/mL) (gray columns) for 18, 24, 48, and 72 hours. Culture supernatants were collected and analyzed by Griess reagent. **Abbreviations:** DEX-P, dexamethasone phosphate; LPS, lipopolysaccharide; NO, nitric oxide.

**Figure 9:**
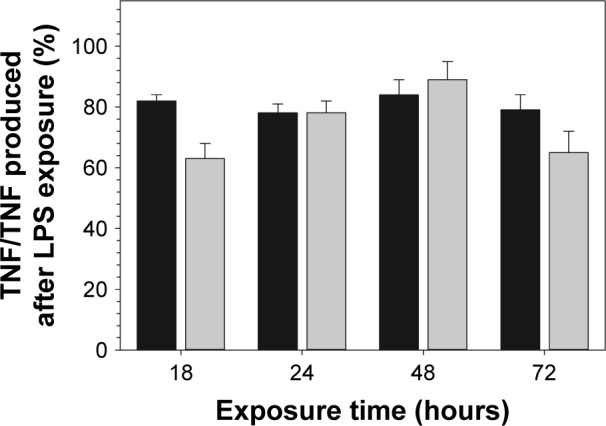
Effect of DEX on TNF-a production (percentage to TNF-a in LPS-only control) of LPS-activated RAW 264.7 macrophages. **Notes:** Cells were exposed to DEX (3.9 µg/mL) from filtered broth collected after the first 24 hours (black columns) of release or DEX-P (DEX equivalent 3.9 µg/mL) (gray columns) for 18, 24, 48, and 72 hours. **Abbreviations:** DEX-P, dexamethasone phosphate; LPS, lipopolysaccharide; TNF-a, tumor necrosis factor-a.

Exposure to DEX-P reduced the amount of NO produced by RAW 264.7 cells stimulated by LPS; similarly, DEX released from the functionalized surfaces decreased NO production (Figure 8). TNF-a data showed a similar inhibitory trend to that observed for NO production (Figure 9).

In general, implants, such as TJR, induce tissue response after implantation into living tissues.^57^ During the inflammatory process, NO and cytokines such as TNF-a are released, and thus, they can be utilized for measuring the level of inflammatory reactions after material implantation, including aseptic periprosthetic osteolysis.^56^ There is a considerable body of evidence showing that there is an increased presence of TNF-a and NO in the fluid and inflamed periprosthetic membrane tissue obtained from loosened implants.^58^–^60^ Because of its pivotal role in anti-inflammatory activity of macrophages, significant effort has been dedicated to developing therapeutic agents that regulate/decrease NO and TNF-a production.

A preliminary study (as shown in the Supplementary material) was performed to determine the optimal DEX concentration capable of reducing the anti-inflammatory activity, and simultaneously not impact cell viability, of RAW 264.7 macrophages. For this purpose, RAW 264.7 macrophages with and without LPS were exposed to a range of DEX concentrations (3.9–100 µg/mL).

As it was observed for DEX alone, DEX released from functionalized TiO_2_ particles obtained by amino route decreased both NO and TNF-a production in LPS-activated macrophages (Figures 8 and 9). Together with results obtained from cellular viability studies (viability >80%) (Figure 7), the anti-inflammatory efficacy of model system reported here was confirmed, excluding the hypothesis of a nonspecific decline in protein synthesis due to drug-induced toxicity. Similar effect was observed in previous studies where the anti-inflammatory compounds tested have shown to simultaneously reduce LPS-induced nitrite accumulation and TNF-a secretion in RAW 264.7 macrophages.^61^ Similar studies have reported the anti-inflammatory effect of DEX released from drug-eluting systems.^51^,^62^

Furthermore, it is important to highlight the biological relevance of this study, which simultaneously focuses on inhibition of different inflammatory mediators NO and TNF-a, and also in contrast to most part of the studies reported in literature, the anti-inflammatory effect was assessed for longer period (ie, 72-hour study instead of 18 hours/24 hours). Hence, from a biological point of view, the results obtained in this study provide a more comprehensive and consistent information about the anti-inflammatory mechanism of the model system developed here; particularly, anti-inflammatory efficacy lasting 3 days as reported in this study suggests that our model system will modulate the acute inflammatory phase (~2–3 days), thus avoiding the unfavorable chronic phase. In addition, DEX released from functionalized particle was added to the cells, just before LPS addition. This observation might be of special importance to elucidate the mechanisms of action of an anti-inflammatory eluting implant, particularly as a protective compound to avoid chronic inflammation. Moreover, despite being released from a cross-linking agent, DEX retained almost the same anti-inflammatory activity; in the amino route, there are two possible bonds that could be hydrolyzed, one is amide bond between the nitrogen of APTES and the succinic anhydride and the other is the ester bond between DEX and the succinic acid. Ester bonds are more easily hydrolyzed than amide ones; thus, it was hypothesized that the release would generate pure DEX molecules; the anti-inflammatory results prove this along with demonstrating the efficacy of our technology.

## Conclusion

Despite being one of the most common complication of TJR, there is no approved therapy to avoid or treat aseptic loosening following wear-induced periprosthetic inflammation/osteolysis. Considerable research has been done to reduce wear debris release and their consequences, including modification on implant design and improvement of surgical techniques, although offering some promise none of these approaches has yet shown to inhibit or reverse particle-induced osteolysis in a human population. This leads to the conclusion that most part of the currently applied therapies depend on the systemic administration of anti-inflammatory agents, meaning that their bioavailability at osteolytic lesions will mainly depend on the limited blood supply at bone–implant interface. Therefore, localized release of anti-inflammatory drugs from implanted TJR could modulate the environment of this host cell/implant interface and related inflammatory events, thus overcoming the aseptic loosening and improving performance of these life-saving devices.

In this study, different routes were applied to develop a drug delivery model system that allows localized and tunable delivery of anti-inflammatory drugs.

Overall, results have also shown that, depending on the synthetic route applied, the DEX-loaded functionalized TiO_2_ particles obtained present different features from size/morphology to drug release profiles.

Furthermore, in vitro studies using RAW 264.7 macrophages indicated that DEX concentrations released from functionalized TiO_2_ particles presented anti-inflammatory activity; particularly, DEX released from surfaces functionalized through the amino route exhibited anti-inflammatory activity comparable with the same concentration of pure DEX.

Therefore, this model system could be a potential approach for patients who undergo TJR to avoid progressive osteolysis and/or to patients who need repeated revision surgery after joint replacement.

## Supplementary materials

Figure S1 Examples of transmission electron microscopy (TEM) images of different surface-modified TiO_2_ particles: (**A**) TiO_2_ particles, (**B**) mercapto-functionalized, (**C**) succinylated mercapto functionalized, and (**D**) dexamethasone-loaded TiO_2_ particles.

Figure S2 Effect of dexamethasone (DEX) on cell viability of RAW 264.7 macrophages exposed to DEX at concentrations between 3.9 and 100 µg/mL for 18 hours, 1 day, 2 days, and 3 days. Cell viability was assessed through MTT (**A**) and LDH assay (**B**).
**Abbreviation:** LDH, lactate dehydrogenase.

Figure S3 Effect of DEX in cell viability of LPS-activated cells. RAW 264.7 macrophages were exposed to DEX at concentrations between 3.9 and 100 µg/mL and also to LPS (1 µg/mL) for 18 hours, 1 day, 2 days, and 3 days. Cell viability was assessed by MTT assay.
**Abbreviations:** DEX, dexamethasone; LPS, lipopolysaccharide.

Figure S4 NO production by RAW 264.7 macrophages upon exposure to LPS (0.5 and 1 µg/mL) for 18, 24, 48, and 72 hours.
**Abbreviations:** LPS, lipopolysaccharide; NO, nitric oxide.

Figure S5 Cellular viability of RAW 264.7 macrophages without (**A**) and with (**B**) LPS (1 µg/mL), after 18, 24, 48, and 72 hours of exposure assessed by MTT assay when different cell densities were utilized.
**Abbreviation:** LPS, lipopolysaccharide.

Figure S6 Effect of different concentrations of acetate buffer utilized for drug release studies, with LPS in cell viability. RAW 264.7 macrophages were exposed to range of acetate buffer (pH =6) concentrations (10%–100%) diluted in DMEM, during 18, 24, 48, and 72 hours. Study performed with 2×10^4^ cells/well + DMEM. **P<0.05, ***P<0.01, ****P<0.001.
**Abbreviations:** DEX, dexamethasone; LPS, lipopolysaccharide.

## Figures and Tables

**Table 1:** Organic content of samples after each step of synthesis as determined through TGA

Synthetic route	Samples	Organic content (%)
	TiO_2_ particles	1.6±0.1
Mercapto	Mercapto-TiO_2_ particles	4.8±0.2
	aCarboxyl-TiO_2_ particles	5.4±0.3
	DEX-TiO_2_ particles conjugated	9.2±0.4
Amino	Amino-TiO_2_ particles	6.0±0.3
	bCarboxyl-TiO_2_ particles	9.2±2.6
	DEX-TiO_2_ particles conjugated	14.8±0.2

**Note:** Carboxyl functionalization was performed via

a pentanoic acid and

b succinic anhydride.

**Abbreviation:** TGA, thermogravimetric analysis.
