# Supplementary figures and images for: Anti-inflammatory drug-eluting implant model system to prevent wear particle-induced periprosthetic osteolysis
Source: Int J Nanomedicine. 2019 Feb 8;14:1069–84. doi: 10.2147/IJN.S188193 (PMC6371946; doi:10.2147/IJN.S188193)

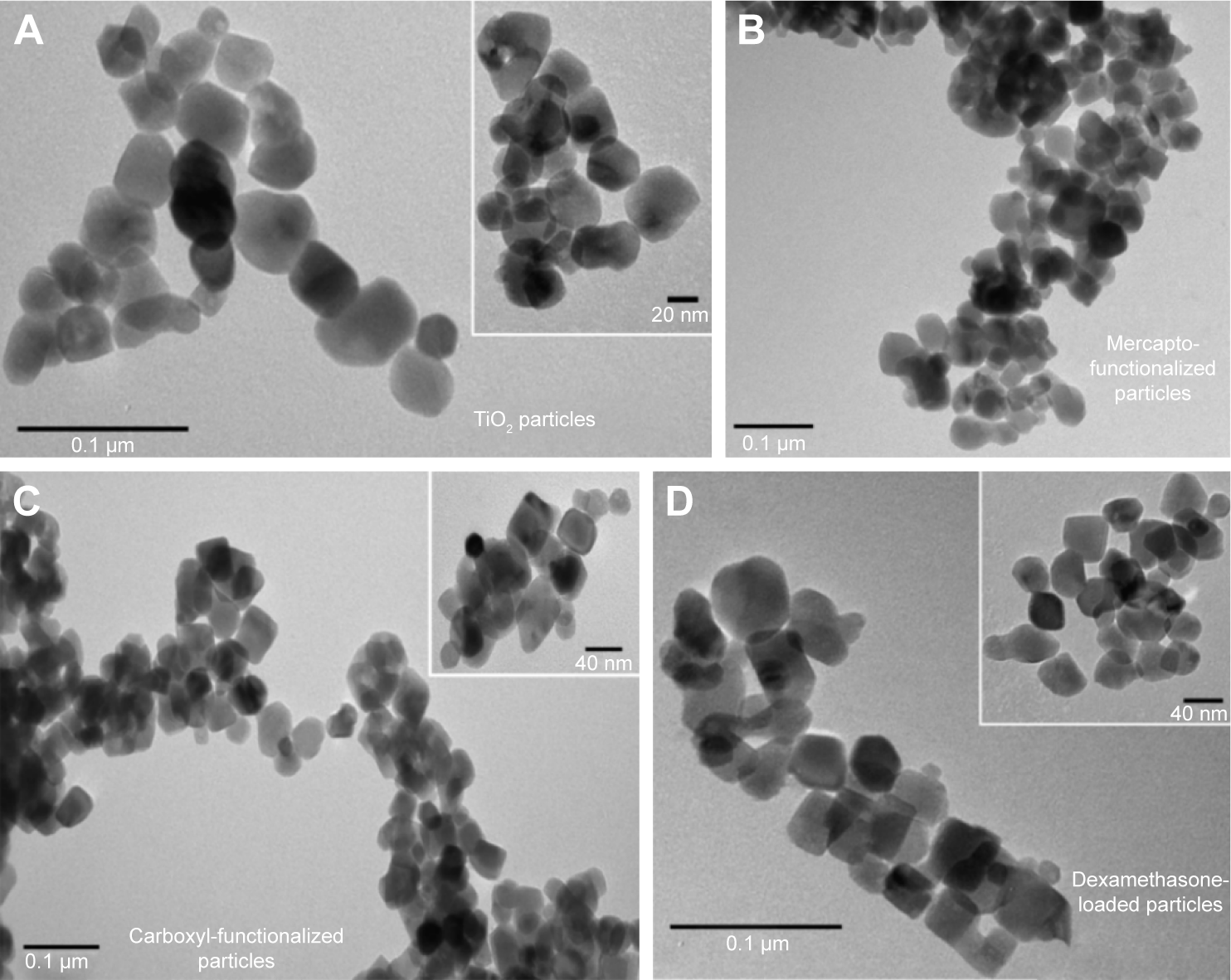

Supplement: Figure S1 — Examples of transmission electron microscopy (TEM) images of different surface-modified TiO2 particles: (A) TiO2 particles, (B) mercapto-functionalized, (C) succinylated mercapto functionalized, and (D) dexamethasone-loaded TiO2 particles. [file ijn-14-1069s1.tif]

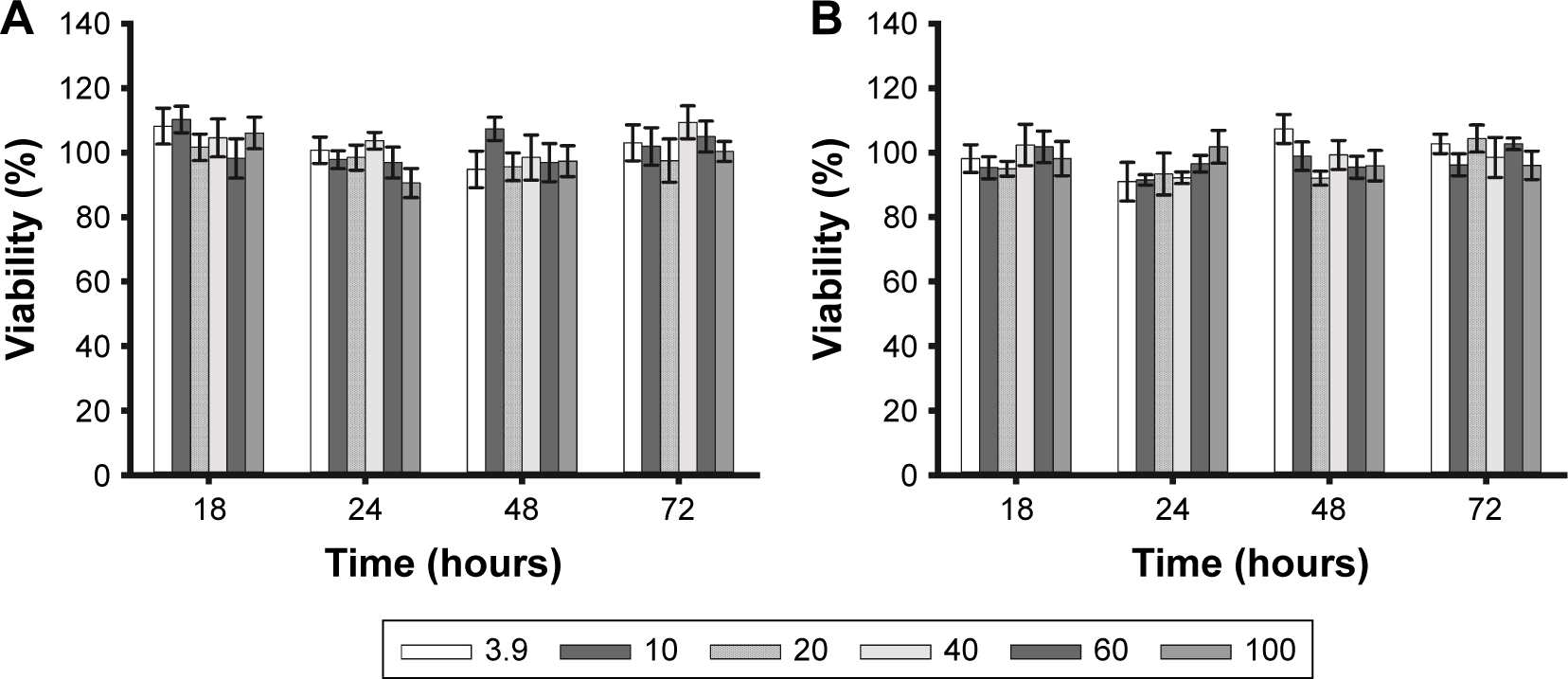

Supplement: Figure S2 — Effect of dexamethasone (DEX) on cell viability of RAW 264.7 macrophages exposed to DEX at concentrations between 3.9 and 100 µg/mL for 18 hours, 1 day, 2 days, and 3 days. Cell viability was assessed through MTT (A) and LDH assay (B). Abbreviation: LDH, lactate dehydrogenase. [file ijn-14-1069s2.tif]

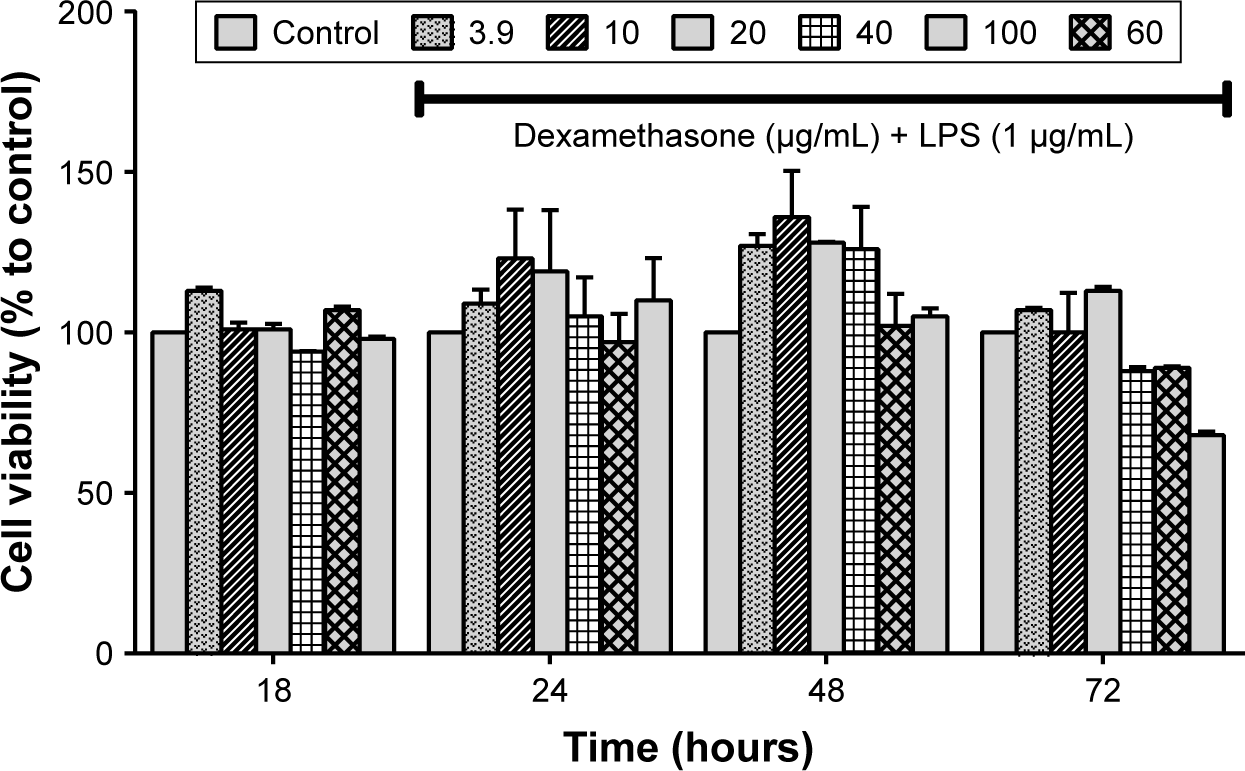

Supplement: Figure S3 — Effect of DEX in cell viability of LPS-activated cells. RAW 264.7 macrophages were exposed to DEX at concentrations between 3.9 and 100 µg/mL and also to LPS (1 µg/mL) for 18 hours, 1 day, 2 days, and 3 days. Cell viability was assessed by MTT assay. Abbreviations: DEX, dexamethasone; LPS, lipopolysaccharide. [file ijn-14-1069s3.tif]

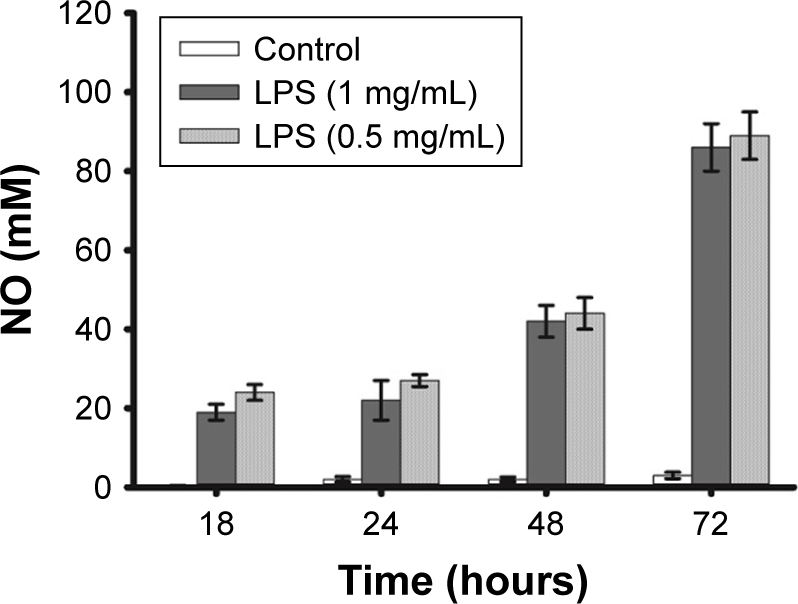

Supplement: Figure S4 — NO production by RAW 264.7 macrophages upon exposure to LPS (0.5 and 1 µg/mL) for 18, 24, 48, and 72 hours. Abbreviations: LPS, lipopolysaccharide; NO, nitric oxide. [file ijn-14-1069s4.tif]

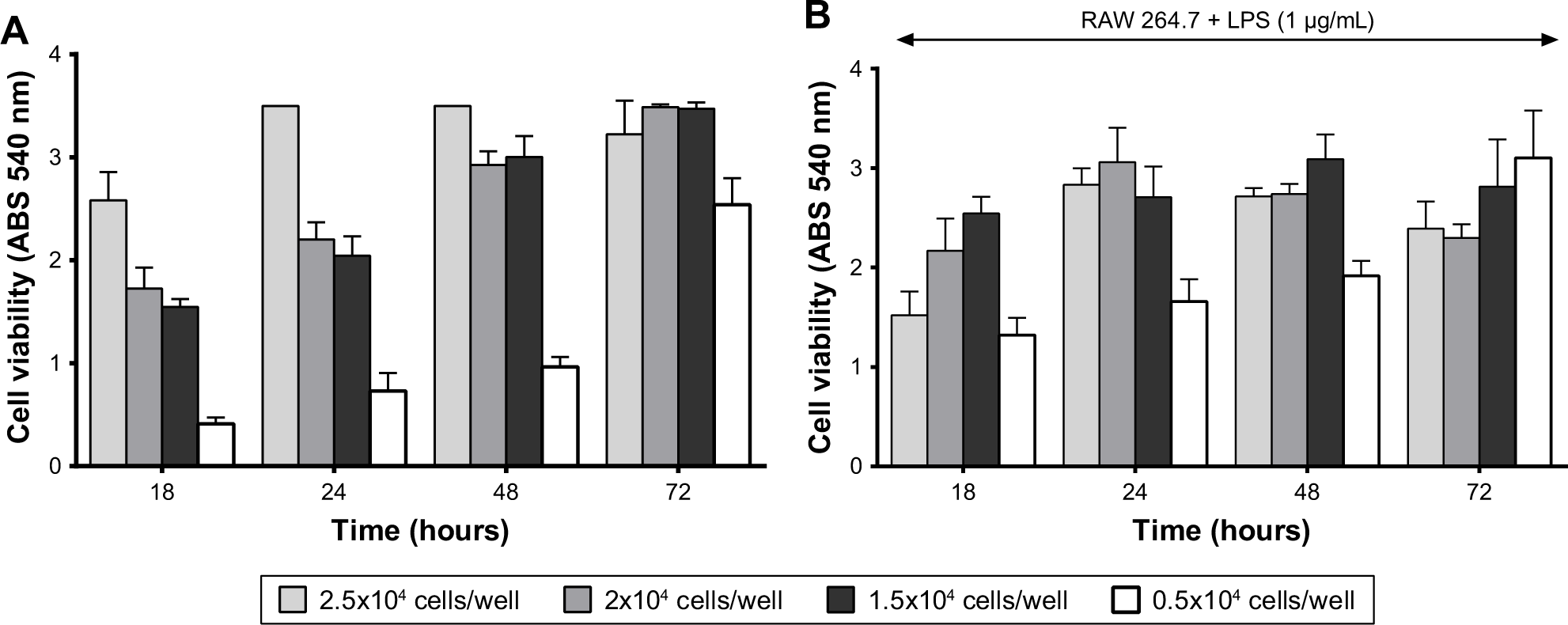

Supplement: Figure S5 — Cellular viability of RAW 264.7 macrophages without (A) and with (B) LPS (1 µg/mL), after 18, 24, 48, and 72 hours of exposure assessed by MTT assay when different cell densities were utilized. Abbreviation: LPS, lipopolysaccharide. [file ijn-14-1069s5.tif]

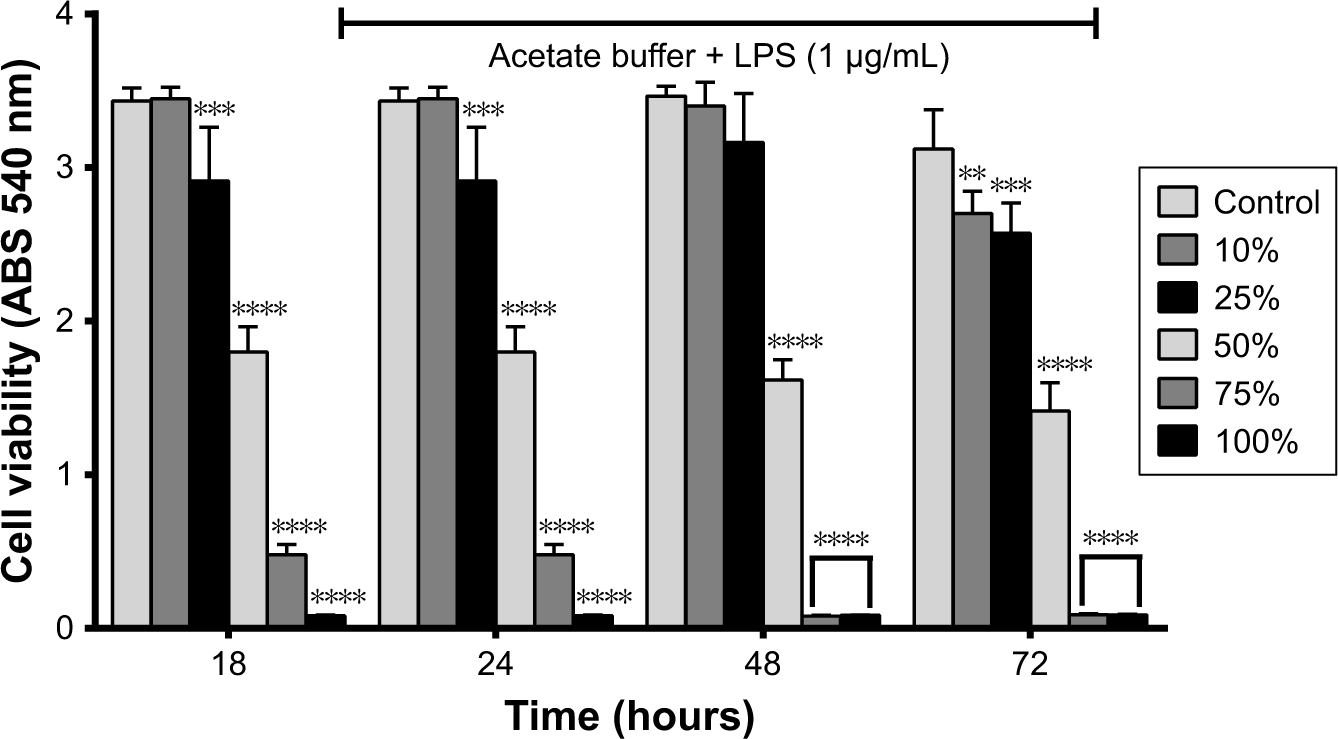

Supplement: Figure S6 — Effect of different concentrations of acetate buffer utilized for drug release studies, with LPS in cell viability. RAW 264.7 macrophages were exposed to range of acetate buffer (pH =6) concentrations (10%–100%) diluted in DMEM, during 18, 24, 48, and 72 hours. Study performed with 2×10^4^ cells/well + DMEM. **P<0.05, ***P<0.01, ****P<0.001. Abbreviations: DEX, dexamethasone; LPS, lipopolysaccharide. [file ijn-14-1069s6.tif]
